# Antipsychotics-related hyperprolactinaemia among patients with schizophrenia in Maiduguri

**DOI:** 10.4102/sajpsychiatry.v30i0.2133

**Published:** 2024-02-01

**Authors:** Falmata B. Shettima, Musa A. Wakil, Taiwo L. Sheikh, Mohammed Abdulaziz, Ibrahim A. Wakawa, Omeiza Beida

**Affiliations:** 1 Department of Mental Health, Faculty of Psychiatry, Federal Neuropsychiatric Hospital, Maiduguri, Nigeria; 2 Department of Mental Health, Faculty of Psychiatry, University of Maiduguri Teaching Hospital, Maiduguri, Nigeria; 3 Department of Mental Health, Faculty of Psychiatry, Ahmadu Bello University Zaria, Kaduna, Nigeria; 4 Department of Mental Health, Faculty of Psychiatry, Federal Neuropsychiatric Hospital Kaduna, Kaduna, Nigeria

**Keywords:** hyperprolactinaemia, antipsychotics, prolactin, schizophrenia, Nigeria

## Abstract

**Background:**

Hyperprolactinaemia among patients on antipsychotic medications is generally overlooked due to lack of outwardly visible symptoms, patient resistance to reporting because the symptoms are perceived as shameful, or to clinician’s insufficient knowledge.

**Aim:**

The study aimed to evaluate the patterns and correlates of hyperprolactinemia among patients with schizophrenia on antipsychotic medications.

**Setting:**

The study was conducted in a psychiatric facility in Maiduguri, Northeastern Nigeria.

**Methods:**

A total of 209 patients with schizophrenia were evaluated through a cross-sectional design and assayed for serum prolactin with ELISA Kits. Frequencies and percentages were tabulated for categorical variables. Variables with significant associations with hyperprolactinaemia on chi-square (*p* < 0.05) were subjected to logistic regression analysis.

**Results:**

The prevalence of hyperprolactinaemia was 45.9% in all patients on antipsychotic medication. The prevalence because of the use of typical and atypical antipsychotics was 51.5% and 25.0%, respectively. Hyperprolactinaemia was significantly associated with typical antipsychotics (β = 0314, *p* = 0.002), high overall drug dosage (β = 2.340, *p* = 0.003), high-dose typical antipsychotics (β = 3.228, *p* = 0.000), twice daily dosing frequency (β = 2.751, *p* = 0.001) and polypharmacy (β = 1.828, *p* = 0.0024).

**Conclusion:**

The findings support that patients on typical, high-dose antipsychotic medications and polypharmacy have a high prevalence of hyperprolactinaemia. As hyperprolactinaemia is often undetectable, screening and patient psycho-education on the significance of the signs and symptoms of hyperprolactinaemia is required for necessary clinical intervention.

**Contribution:**

The study provides evidence for the rational use of antipsychotic medications in sub-Saharan Africa.

## Introduction

The hormone prolactin is secreted by the anterior pituitary gland, and its release is mainly under the control of dopamine (prolactin inhibitory factor) in the hypothalamic-pituitary axis and an increase in dopamine availability inhibits prolactin production, while a blockade of dopamine (D2 receptors) leads to increased prolactin production.^1,2^ Hyperprolactinaemia is defined as ‘fasting levels of plasma prolactin at least 2 h after waking above 20 ng/mL in men and above 25 ng/mL in women’.^3^ Several physiologic (sleep, stress, pregnancy and lactation), pathologic (brain diseases such as pituitary adenomas and systemic diseases such as hypothyroidism) and pharmacologic states including psychotropic medications can result in increased plasma prolactin levels.^2,3,4^ As dopamine inhibits prolactin release in the pituitary gland, antipsychotic medications that decrease dopaminergic activity will lead to an increase in serum prolactin level.^1^

Hyperprolactinaemia has been observed among patients on antipsychotic medications with greater D2 receptor binding, especially those on typical antipsychotics.^5,6^ In most cases, the atypical antipsychotics have a lower propensity to increase prolactin levels than typical antipsychotics with clozapine, olanzapine and quetiapine only mildly and transiently elevating prolactin levels while risperidone and amisulpride with greater affinity to D2 receptors causing a marked and sustained increase in serum prolactin levels.^7,8^ In addition, the tendency of antipsychotics to elevate plasma prolactin level is dose dependent with higher doses more likely to result in high prolactin levels.^9,10^ Dopamine D2 receptor occupancy by antipsychotic medications at 65% predicts therapeutic response, while hyperprolactinaemia becomes manifest at 72% occupancy.^11^

The prevalence of hyperprolactinaemia among patients with schizophrenia on antipsychotic medications has been estimated to range between 30% and 75%.^12,13,14^ It is more frequent among women than men even among drug-naïve first-episode psychosis patients, higher doses of medications, greater severity of illness on the Positive and Negative Symptoms Scale (PANSS) or Brief Psychiatric Rating Scale (BPRS) and the newly initiated on antipsychotics.^14,15,16^ Hyperprolactinaemia has also been observed to be significantly higher in drug-naïve patients than in healthy controls.^17,18^ Hyperprolactinaemia may be asymptomatic in some patients and the most common symptoms of chronic hyperprolactinemia are reproductive dysfunction (anovulatory cycles, menstrual irregularity, sub-fertility, decreased oestrogen and testosterone production), sexual impairment (diminished libido, erectile dysfunction, retrograde or painful ejaculation, orgasmic dysfunction), breast abnormalities (breast enlargement, galactorrhoea) and bone impairment (decreased bone mineral density and osteoporosis).^1,4^

The only prior study on the prevalence and correlates of hyperprolactinaemia in Nigeria was carried out in the southern part and only explored on the relationship of hyperprolactinaemia with medication class (typical or atypical) and medication dosage and found that the greatest predictor of hyperprolactinaemia was the medication dosage.^12^ Even though the majority of patients with schizophrenia in northeastern Nigeria because of their poor socio-economic status are treated at the facility with typical antipsychotic medications that pose a greater risk of hyperprolactinaemia, there are no studies on the prevalence and correlates of hyperprolactinaemia among these patients. The study seeks to evaluate patterns of hyperprolactinaemia among patients on typical and atypical antipsychotic medications. Aside from adding to the existing body of knowledge on psychopharmacology in West Africa, the outcome of this study will help to improve the management strategy of patients on antipsychotic medications.

## Research methods and design

### Study setting

The study was conducted at Federal Neuro-Psychiatric Hospital, Maiduguri. The hospital is a tertiary centre that is located along Baga Road. It serves as a referral centre from neighbouring countries of Chad, Niger and Cameroun.

### Study population

A total of 209 patients with schizophrenia completed the study. The inclusion criteria were participants between ages 18 and 65 years with an International Classification of disease (ICD-10) diagnosis of schizophrenia who have been attending the clinic for at least 6 months and have given informed consent in writing. Exclusion criteria included pregnant and lactating women and the presence of endocrinological disorders such as hypothyroidism, pituitary gland disease and diabetes mellitus.

### Study design and procedure

The study was cross-sectional in design. Each patient was interviewed in a private and well-secured room to assure privacy, confidentiality and safety and the administration of the questionnaires took on average about 45 min to complete. The questionnaires have been validated for use in Nigeria.

### Study measures

A socio-demographic questionnaire requesting information about the participants’ age, gender, educational status, marital status, occupational status, type, dosage and frequency of antipsychotic medications was administered. A single venous blood sample was collected for serum prolactin analysis. The severity of symptoms was measured with PANSS and the medication adherence with Medication Adherence Rating Scale (MARS).

### Methods for serum prolactin measurements

Blood samples were taken at least 2 h after the last meal and 30 min of rest. Venous blood was extracted for centrifugation in a glass tube, and the plasma was stored in a plastic tube at –80°C. Prolactin concentration was assayed using the Enzyme Immunoassay Test Kit [Microparticle enzyme immunoassay (MEIA)] in the serum. The prolactin hormone (PRL) AccuBind^TM^ELISA Test kit was used to quantitatively measure prolactin in the serum of patients with schizophrenia. The kit is a rapid, sensitive and reliable assay for the measurement of prolactin level based on a principle of a solid-phase enzyme-linked immunosorbent assay.^19^ The normal ranges of values are 1.2–19.5 and 1.8–18.5 in adult females and males, respectively.^19^ Assessments of serum prolactin levels were performed at the Department of Chemical Pathology, University of Maiduguri Teaching Hospital.

### Positive and Negative Symptoms Scale

The PANSS is used to measure the severity of illness among patients with schizophrenia. It is a clinician-based interview that takes approximately 45 min to complete, rated on a 7-point scale ranging from 1 (no symptoms) to 7 (extreme symptoms).^20^ The scale has 30 items with 7 items each measuring positive and negative symptoms and 16 items measuring general psychopathology.^20^ The scale has good psychometric properties and has been previously used in Nigeria.^21^

### Medication Adherence Rating Scale

It is a 10-item self-reporting dimensional scale designed to measure drug-taking behaviour with a dichotomised response option of yes or no.^22^ It comprises both positively and negatively worded drug-taking behaviour statements to limit social desirability bias. A response consistent with non-adherence is coded as 0, while a response consistent with adherence is coded as 1. A no response indicative of adherence is coded as 1 for questions 1–6 and 9–10 while a yes response indicative of adherence is coded as 1 for questions 7 and 8. Items 1–4 assess medication adherence behaviour, items 5–8 assess attitudes towards taking medication and items 9–10 assess negative medication side effects and attitude towards psychotropic medications. Total score ranges from 0 (low) to 10 (high) medication adherence. The scale has good internal consistency (alpha = 0.75) and has been validated for use in Nigeria.^22,23^

### Statistical analysis

Frequencies and percentages were used for descriptive analysis. A chi-square test was used to analyse the association between socio-clinical factors and prolactin levels. Variables with significant associations were subjected to binary logistic regression analysis. All tests were two tailed with the significance level set at 0.05. SPSS version 18 was used for data entry and analysis.

### Ethical considerations

The study was registered with the National Health Research Ethics Committee (NHREC/06/01/2020) and ethical approval was obtained from the Research Ethics Committee of Federal Neuro-psychiatric Hospital Maiduguri. Patients with written informed consent with an established diagnosis of schizophrenia, schizoaffective disorder and delusional disorder using ICD-10 diagnostic criteria participated in the study (FNPH/072021/REC133). Only those who consented in writing were interviewed. To ensure privacy, only codes were used as means of identification.

## Results

The mean prolactin level was 32.24 ± 44.02. The mean prolactin levels for typical and atypical antipsychotics were 35.17 ± 45.29 and 21.26 ± 37.37, respectively, and the mean levels for males and females were 27.91 ± 36.30 and 37.75 ± 51.92, respectively. Close to 60% of the population were between the ages of 25–34 and 35–44 years. Males constituted 56% of the population, 70.3% were unemployed, 78.9% were on typical antipsychotic medications and 54.1% had high prolactin levels as presented in Table 1.

**TABLE 1:** Socio-clinical characteristics of the study participants (*n* = 209).

Variables	*n*	%	Mean
**Age (years)**			
18–24	20	9.6	-
25–34	70	33.5	-
35–44	53	25.4	-
45–54	40	19.1	-
55–65	26	12.4	-
**Gender**			
Male	117	56.0	-
Female	92	44.0	-
**Occupational status**			
Employed	62	29.7	-
Unemployed	147	70.3	-
**Duration of treatment**			
< 1 year	21	10.0	-
1–5 years	93	44.5	-
5–10 years	54	25.8	-
> 10 years	41	19.6	-
**MARS**			
Low adherence	90	43.1	-
Medium adherence	92	44.0	-
High adherence	27	12.9	-
**Number of drugs**			
1	85	40.7	-
2	119	56.9	-
3 or more	5	2.4	-
**Dosing frequency**			
1	127	60.8	-
2	82	39.2	-
**Drug type**			
Typical	165	78.9	-
Atypical	44	21.1	-
**PANSS score**			
Normal	98	46.9	-
Mildly ill	73	34.9	-
Moderately ill	29	13.9	-
Severely ill	9	4.3	-
**Prolactin**			
Normal	113	54.1	-
High	96	45.9	-
**Mean prolactin level by gender**			
Total	-	-	32.24 ± 44.02
Males	-	-	27.91 ± 36.30
Females	-	-	37.75 ± 51.92
**Mean prolactin level by medication type**			
Total	-	-	32.24 ± 44.02
Typical APM	-	-	35.17 ± 45.29
Atypical APM	-	-	21.26 ± 37.37

Typical (haloperidol + trifluoperazine + chlorpromazine), atypical (olanzapine + risperidone + clozapine), APM (antipsychotic medication), drug categories under number of drugs (antipsychotics, anticholinergics, mood stabilisers). MARS, Medication Adherence Rating Scale; PANSS, Positive and Negative Symptoms Scale.

**FIGURE 1:**
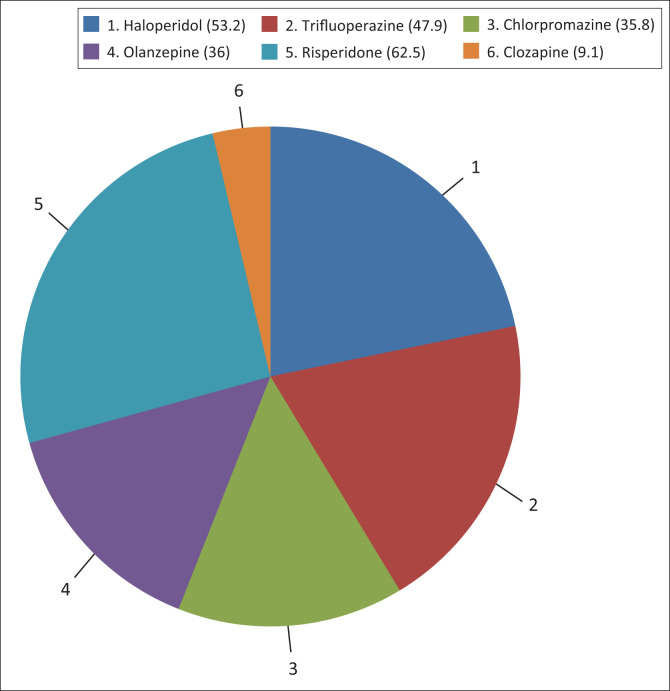
Frequency of hyperprolactinemia according to specific drug type (χ^2^ = 12.11, *р* = 0.033).

The significant differences in the frequency of hyperprolactinaemia according to drug types are shown in Figure 1 (χ^2^ =12.11, *р* = 0.033), and Table 2 shows that none of the socio-demographic variables were associated with hyperprolactinaemia. The clinical variables of significance were the type of medications, dosage of medication, number of drugs and frequency of dosing. Hyperprolactinaemia was observed among 51.5% of those on typical antipsychotics compared with 25% of those on atypical antipsychotics (χ^2^ = 9.834, *p* = 0.002), 56.1% of those on high medication dosage (χ^2^ = 9.080, *p* = 0.003) compared with 35.3% on low dose, 61.0% of those on twice daily dosing frequency (χ^2^ = 12.30, *p* = 0.000) compared with 36.2% once-daily dosing frequency and with use of two or more drugs (χ^2^ = 6.896, *p* = 0.0032) compared with one drug. All the significant variables subjected to logistic regression analysis remained statistically significant (Table 3).

**TABLE 2:** Socio-clinical correlates of hyperprolactinaemia.

Variables	Normal prolactin		Elevated prolactin		χ^2^	*P*
	*n*	%	*n*	%		
**Age (years)**						
18–24	9	45.0	11	55.0	4.852	0.303
25–34	33	47.1	37	52.9	-	-
35–44	31	58.5	22	41.5	-	-
45–54	22	55.0	18	45.0	-	-
55–65	18	30.8	8	30.8	-	-
**Gender**						
Male	65	55.6	52	44.4	0.237	0.626
Female	48	52.2	44	47.8	-	-
**Occupation status**						
Employed	37	59.7	25	40.3	1.117	0.291
Unemployed	76	51.7	71	48.3	-	-
**Duration of treatment**						
< 1 year	8	38.1	13	61.9	6.282	0.099
1–5 years	51	54.8	42	45.2	-	-
5–10 years	26	48.3	28	51.9	-	-
> 10 years	28	55.6	13	31.7	-	-
**PANSS score**						
Normal	54	55.1	44	44.9	0.457	0.928
Mildly ill	40	54.8	33	45.2	-	-
Moderately ill	14	48.3	15	51.7	-	-
Severely ill	5	55.6	4	44.4	-	-
**Medication type**						
Typical	80	48.5	85	51.5	9.834	0.002*
Atypical	33	75.0	11	25.0	-	-
**Typical**						
Low dose	52	63.4	30	36.6	13.38	0.000*
High dose	29	34.9	54	65.1	-	-
**Atypical**						
Low dose	13	68.4	6	31.6	0.313	0.576
High dose	19	76.0	6	24.0	-	-
**Overall dosage**						
Low dose	66	64.7	36	35.3	9.080	0.003*
High dose	47	43.9	60	56.1	-	-
**Number of drugs**						
1	55	64.7	30	35.3	6.896	0.032*
2	55	46.2	64	53.8	-	-
3 or more	3	60.0	2	40.0	-	-
**Dosing frequency**						
Once	81	63.8	46	36.2	12.30	0.000*
Twice	32	39.0	50	61.0	-	-
**MARS**						
Low adherence	9	33.3	18	66.7	5.586	0.061
Medium adherence	51	55.4	41	44.6	-	-
High adherence	53	58.9	37	41.4	-	-

* , significant association.

Note: Typical (haloperidol + Trifluoperazine + chlorpromazine), atypical (olanzapine + risperidone + clozapine). All the antipsychotics used were converted to their chlorpromazine equivalent dose. Typical low dose (≤300 mg chlorpromazineeuivalent), typical high dose (> 300 mg chlorpromazine equivalent), atypical low dose (≤ 300 mg chlorpromazine equivalent), atypical high dose (> 300 mg chlorpromazine equivalent). MARS, Medication Adherence Rating Scale; PANSS, Positive and Negative Symptoms Scale.

**TABLE 3:** Logistic regression analysis.

Variables	EXP (B)	*P*
**Type of medication**	0.314	0.002*
**Typical dosage**	3.228	0.000*
**Overall dosage**	2.340	0.003*
**Number of drugs**	1.828	0.024*
**Dosing frequency**	2.751	0.001*

* , significant association.

## Discussion

The study showed that 45.9% of patients with schizophrenia on antipsychotic medications had hyperprolactinaemia. This study found a higher prevalence of hyperprolactinaemia (45.9%) than a previous report from soutrhern Nigeria, which found a frequency of 30%. The reason for the differences could be due to variations in sample size, patient characteristics and treatment length, as prolactin was measured only 8 weeks after starting antipsychotic medications in drug naive individuals in their study. Theprevalence of hyperprolactinaemia in this study is higher than previous report in southern Nigeria of 30%.^12^ This may be because of the differences in sample size, patient characteristics and duration of treatment, as prolactin was assessed at only 8 weeks from the time of commencement of antipsychotic medications in drug naïve patients. In general, hyperprolactinaemia may take between 7 and 75 days from commencing antipsychotics.^8,24^ Our findings on the prevalence of hyperprolactinaemia are comparable to studies carried out in China, (55.9%)^15^ Spain, (52.4%)^13^ Saudi Arabia (44.3%)^25^ and Japan, (53.8%).^26^ This study did not find any relationship between socio-demographic factors and hyperprolactinaemia. While there was no statistically significant gender difference in hyperprolactinaemia among our study participants, females had a significantly higher mean prolactin level than males. Many previous studies have found a significantly higher prevalence of hyperprolactinaemia among females than males with the differences regarded by the ability of oestrogen to raise prolactin levels through enhancement of lactotropic cell responsiveness to prolactin-releasing stimuli.^7,27^ However, other studies have failed to establish any gender differences in hyperprolactinaemia.^25,26^

In keeping with current literature, this study showed a significant association between hyperprolactinaemia and antipsychotic medications with a twofold difference in the prevalence of hyperprolactinaemia among patients placed on typical antipsychotics (51.5%) compared with atypical antipsychotics (25%). Furthermore, risperidone had the greatest increase in prolactin levels (62.5%), followed by haloperidol (57.3%) and least with clozapine (9.1%). The finding is consistent with prior randomised longitudinal double-blind clinical trials and cross-sectional studies showing greater effects of haloperidol and risperidone on prolactin levels than other antipsychotic medications.^2,28,29^ When individuals on typical antipsychotics were categorised into high (> 300 mg equivalent of chlorpromazine) and low-dose, hyperprolactinaemia was almost two times more prevalent among individuals on high-dose (*p* = 0.000) than those on low dose. This is consistent with the accumulating evidence of the antipsychotic dose-dependent proneness for hyperprolactinaemia.^15^ Additionally, individuals on twice daily dosing frequency of medication and individuals on two or more drugs had a significantly higher frequency of hyperprolactinaemia. These relationships may be directly related to the drug dose. As the number of drugs and frequency of dosing increases, there is a possibility that the overall medication dosage increases, thereby increasing the tendency for hyperprolactinaemia. The finding on the relationship between number of drugs and hyperprolactinaemia has been reported by Alosaimi et al.,^25^ who reported that almost half of the patients on two or more drugs had hyperprolactinaemia compared with patients on one drug (*p* = 0.03).

As with prior studies,^6,7^ the substantial variability in the prolactin levels of the various antipsychotic medications examined in this study demonstrates their differential D2 receptor-binding affinity. Furthermore, variability in the ratio of D2 receptor occupancy in the pituitary gland relative to the brain by antipsychotic medications may account for the differences in prolactin level.^7^ Antipsychotic medications, especially risperidone and typical antipsychotics that poorly cross the blood–brain barrier, caused a relatively higher ratio of D2 receptor occupancy in the pituitary gland relative to the brain.^3,11,30^ The antagonism of dopamine receptor reduces the level of dopamine in the anterior pituitary, and this results in the loss of the inhibitory effect of dopamine on prolactin in the lactotroph cells thereby increasing the tendency for hyperprolactinaemia.^31^ Therefore, antipsychotic medications with lower potentials for hyperprolactinaemia should be considered when prescribing an antipsychotics. Prolactin should be measured before commencement of antipsychotic medications; it should be measured regularly alongside regular clinical evaluation of signs and symptoms of hyperprolactinaemia.

The study has several strengths, namely, it is the first study to examine the prevalence and associated risk factors of hyperprolactinaemia in North-Eastern Nigeria in a fairly modest sample size. The study further explored the prevalence of hyperprolactinaemia of the commonly available antipsychotics. Some of the limitations include its cross-sectional nature making it difficult to establish causality and no baseline prolactin measurement. Pituitary gland disease and hypothyroidism were only ruled out; through clinical evaluation and not radiologically using computed tomography and magnetic resonance imaging, which are the gold standard. Symptoms of hyperprolactinaemia were not explored; thus individuals with hyperprolactinaemia with and without symptoms were not distinguished. The skewed distribution of typical over atypical antipsychotics is a limitation of this study. Another possible limitation is that based on the low socio-economic status of the majority of the study population, it may not be feasible to routinely measure serum prolactin level in all patients.

## Conclusion

We found a high prevalence of hyperprolactinaemia among patients with schizophrenia on antipsychotic medications. Therefore, measurement of serum prolactin level should routinely be incorporated among patients placed on antipsychotics especially those on prolactin-raising antipsychotics, high doses of medications, polypharmacy and more than once daily dosing of medication to optimise adherence and improve quality of life.

## References

[1] Milano W, D’Acunto CW, De Rosa M, et al. Recent clinical aspects of hyperprolactinemia induced by antipsychotics. Rev Recent Clin Trials. 2011 Jan 1;6(1):52–64. 10.2174/157488711793980138 20868350

[2] Dehelean L, Romosan AM, Papava I, et al. Prolactin response to antipsychotics: An inpatient study. PLoS One. 2020 Feb 4;15(2):e0228648. 10.1371/journal.pone.0228648 32017792 PMC6999917

[3] Ajmal A, Joffe H, Nachtigall LB. Psychotropic-induced hyperprolactinemia: A clinical review. Psychosomatics. 2014 Jan 1;55(1):29–36. 10.1016/j.psym.2013.08.008 24140188

[4] Milano W, Colletti C, Capasso A. Hyperprolactinemia induced by antipsychotics: From diagnosis to treatment approach. Endocr Metab Immune Disord Drug Targets. 2017 Mar 1;17(1):38–55. 10.2174/1871530317666170424102332 28440197

[5] Ates MA, Tutuncu R, Oner I, et al. Relationship between plasma levels of prolactin and the severity of negative symptoms in patients with schizophrenia. Klin Psikofarmakol B. 2015 Mar 1;25(1):27–37. 10.5455/bcp.20141212113905

[6] Kapur S, Zipursky R, Jones C, Remington G, Houle S. Relationship between dopamine D2 occupancy, clinical response, and side effects: A double-blind PET study of first-episode schizophrenia. Am J Psychiatry. 2000 Apr 1;157(4):514–520. 10.1176/appi.ajp.157.4.514 10739409

[7] Peuskens J, Pani L, Detraux J, De Hert M. The effects of novel and newly approved antipsychotics on serum prolactin levels: A comprehensive review. CNS Drugs. 2014 May;28(5):421–453. 10.1007/s40263-014-0157-3 24677189 PMC4022988

[8] Lally J, Ajnakina O, Stubbs B, et al. Hyperprolactinaemia in first episode psychosis – A longitudinal assessment. Schizophr Res. 2017 Nov 1;189:117–125. 10.1016/j.schres.2017.07.037 28755878

[9] Haddad PM, Wieck A. Antipsychotic-induced hyperprolactinaemia: Mechanisms, clinical features and management. Drugs. 2004 Oct;64(20):2291–2314. 10.2165/00003495-200464200-00003 15456328

[10] Yoshida K, Takeuchi H. Dose-dependent effects of antipsychotics on efficacy and adverse effects in schizophrenia. Behav Brain Res. 2021 Mar 26;402:113098. 10.1016/j.bbr.2020.113098 33417992

[11] Arakawa R, Okumura M, Ito H, et al. Positron emission tomography measurement of dopamine D2 receptor occupancy in the pituitary and cerebral cortex: Relation to antipsychotic-induced hyperprolactinemia. J Clin Psychiatry. 2010 Feb 23;71(9):14740. 10.4088/JCP.08m04307yel 20361897

[12] Olose EO, Chukwujekwu CD, Busari CO, Igwe MN. Hyperprolactinemia in psychiatric patients taking antipsychotic medications: A longitudinal study in a tertiary hospital in South-South, Nigeria. Biol Med. 2021;13(9):100–292.

[13] Bonete Llacer JM, Martinez Hortelano A, Richart Albelda B. Hyperprolactinemia in psychotic patients treated in monotherapy with long-acting injectable antipsychotics. Int J Psychiatry Clin Pract. 2019 Jul 3;23(3):189–193. 10.1080/13651501.2019.1576905 30848967

[14] Herceg M. Correlation between prolactin and symptom profile in acute admitted women with recurrent schizophrenia. Psychiatr Danub. 2020;32(3–4):367–372. 10.24869/psyd.2020.367 33370734

[15] Wang ZM, Xiang YT, An FR, et al. Frequency of hyperprolactinemia and its associations with demographic and clinical characteristics and antipsychotic medications in psychiatric inpatients in China. Perspect Psychiatr Care. 2014 Oct;50(4):257–263. 10.1111/ppc.12050 24256051

[16] Delgado-Alvarado M, Tordesillas-Gutierrez D, Ayesa-Arriola R, et al. Plasma prolactin levels are associated with the severity of illness in drug-naive first-episode psychosis female patients. Arch Womens Ment Health. 2019 Jun;22(3):367–373. 10.1007/s00737-018-0899-x 30097769

[17] Del Cacho N, Butjosa A, Vila-Badia R, et al. Prolactin levels in drug-naïve first-episode nonaffective psychosis patients compared with healthy controls. Sex differences. Psychiatry Res. 2019 Jun 1;276:218–222. 10.1016/j.psychres.2019.03.027 31112855

[18] Wasnik V, Khess CR, Munda S, Bijali S. Serum prolactin level and its correlation with psychopathology in drug free/drug naive schizophrenia a case control study. Asian J Psychiatry. 2019 Jan 1;39:1–5. 10.1016/j.ajp.2018.11.002 30453151

[19] Tietz N. Clinical guide to laboratory test. 2nd ed. Philadelphia, PA: WB Saunders; 1992.

[20] Kay SR, Fiszbein A, Opler LA. The Positive and Negative Syndrome Scale (PANSS) for schizophrenia. Schizophr Bull. 1987;13(2):261–76. 10.1093/schbul/13.2.261 3616518

[21] Odinka PC, Ndukuba AC, Muomah RC, et al. Positive and negative symptoms of schizophrenia as correlates of help-seeking behaviour and the duration of untreated psychosis in South-East Nigeria. S Afr J Psychiatry. 2014;20(4):166. 10.4102/sajpsychiatry.v20i4.536

[22] Thompson K, Kulkarni J, Sergejew AA. Reliability and validity of a new Medication Adherence Rating Scale (MARS) for the psychoses. Schizophr Res. 2000;42(3):241–247. 10.1016/S0920-9964(99)00130-9 10785582

[23] Owie GO, Olotu SO, James BO. Reliability and validity of the Medication Adherence Rating Scale in a cohort of patients with schizophrenia from Nigeria. Trends Psychiatry Psychother. 2018 May 14;40(2):85–92. 10.1590/2237-6089-2017-0077 29768528

[24] Kelly DL, Wehring HJ, Earl AK, et al. Treating symptomatic hyperprolactinemia in women with schizophrenia: Presentation of the ongoing DAAMSEL clinical trial (Dopamine partial Agonist, Aripiprazole, for the Management of Symptomatic ELevated prolactin). BMC Psychiatry. 2013;13:214. 10.1186/1471-244X-13-214 23968123 PMC3766216

[25] Alosaimi FD, Fallata EO, Abalhassan M, et al. Prevalence and risk factors of hyperprolactinemia among patients with various psychiatric diagnoses and medications. Int J Psychiatry Clin Pract. 2018 Oct 2;22(4):274–281. 10.1080/13651501.2018.1425459 29334291

[26] Kikuchi T, Iwamoto K, Sasada K, Aleksic B, Yoshida K, Ozaki N. Sexual dysfunction and hyperprolactinemia in Japanese schizophrenic patients taking antipsychotics. Prog Neuropsychopharmacol Biol Psychiatry. 2012 Apr 27;37(1):26–32. 10.1016/j.pnpbp.2011.11.016 22172534

[27] Retief M, Chiliza B, Phahladira L, Emsley R, Asmal L. Prolactin, flupenthixol decanoate and first episode schizophrenia–clinical and laboratory correlates. Metab Brain Dis. 2019 Dec;34(6):1679–1687. 10.1007/s11011-019-00474-5 31422510

[28] Volavka J, Czobor P, Cooper TB, et al. Prolactin levels in schizophrenia and schizoaffective disorder patients treated with clozapine, olanzapine, risperidone, or haloperidol. J Clin Psychiatry. 2004 Jan 1;65(1):57–61. 10.4088/JCP.v65n0109 14744169

[29] Melkersson K. Differences in prolactin elevation and related symptoms of atypical antipsychoties in schizophrenic patients. J Clin Psychiatry. 2005 Jun 1;66(6):761–767. 10.4088/JCP.v66n0614 15960571

[30] Stojkovic M, Radmanovic B, Jovanovic M, Janjic V, Muric N, Ristic DI. Risperidone induced hyperprolactinemia: From basic to clinical studies. Front Psychiatry. 2022;13:874705. 10.3389/fpsyt.2022.874705 35599770 PMC9121093

[31] Holt RI, Peveler RC. Antipsychotics and hyperprolactinaemia: Mechanisms, consequences and management. Clin Endocrinol. 2011 Feb;74(2):141–147. 10.1111/j.1365-2265.2010.03814.x 20455888

